# Association of Masticatory Efficiency and Reduced Number of Antagonistic Contacts Due to Extraction, Changing Dentition or Malocclusion in Children

**DOI:** 10.3390/dj11030064

**Published:** 2023-02-28

**Authors:** Odri Cicvaric, Renata Grzic, Marija Simunovic Erpusina, Suncana Simonic-Kocijan, Danko Bakarcic, Natasa Ivancic Jokic

**Affiliations:** 1 Department of Paediatric Dentistry, Faculty of Dental Medicine, University of Rijeka, 51000 Rijeka, Croatia; 2 Department of Prosthodontics, Faculty of Dental Medicine, University of Rijeka, 51000 Rijeka, Croatia

**Keywords:** dental occlusion, malocclusion, mastication, particle size, tooth extraction

## Abstract

Background: Tooth extraction, changing dentition and malocclusion can decrease area of occlusal contact and negatively affect masticatory efficiency. Aim of this study was to evaluate difference in masticatory efficiency in association with previously named factors. Materials and methods: In this cross-sectional study masticatory efficiency parameters (number of particles, mean diameter and mean surface of particles) determined with optical scanning method were compared between children with healthy dentition (12 girls, 12 boys, age 3 to 14) and children with lost antagonistic contacts due to tooth extraction, changing dentition and malocclusions (12 girls, 12 boys, age 3 to 14). Results: Number of chewed particles is significantly higher in a group of children with healthy dentition (*p* < 0.001), and chewed particles’ mean diameter and surface are significantly higher in the Group 2 (*p* < 0.001; *p* < 0.001). Number of lost occlusal contacts is not in correlation with masticatory efficiency parameters (*p*= 0.464; *p*= 0.483; *p*= 0.489). Conclusions: Children with lost antagonistic contacts have an impaired masticatory efficiency in comparison to children with complete dentition, but there is no difference regarding the aetiology of contact loss.

## 1. Introduction

One of the basic prerequisites for oral and general health is the masticatory function [1]. The greatest importance of mastication is manifested in proper nutrition and intake of essential and non-essential nutrients. Nutrition affects the homeostasis of the organism and can increase or decrease the risk of various systemic diseases [2,3]. Furthermore, the adequate masticatory function is important for theproper growth and development of orofacial structures and it reduces the chance of the development of orthodontic anomalies [2,3,4,5]. The optimal masticatory function also, directly and indirectly, affects speech development [1,6].

The mastication apparatus as a part of the stomatognathic system consists of four different tissues, four major components: bones, muscles, teeth, and soft tissues. With mastication begins a complex process of processing food, that is, food is prepared for swallowing, digestion, and absorption of nutrients. It implies a combination of cyclical and learned reflex jaw movements that enable the mechanical processing, crushing of food. During the act of chewing, forces are transferred from the teeth to the food bolus, whereby mobile soft tissues (the tongue, cheeks, and lips) and facial and masticatory muscles position the food bolus between the teeth maximizing the mechanical breakage of the food. Muscle growth, strength, and coordination are needed not just for positioning the food bolus between teeth but for applying forces through bones (upper and lower jaw) and teeth onto the food bolus. Alveolar processes of jaws provide anchorage for erupted teeth which come in contact with food and transfer the muscle forces. The different occlusal surface of different teeth ensures the right direction of forces for the breakage of food particles that are dissolved and moistened with saliva, forming the appropriate food bolus. Saliva also contains enzymes for food decomposition. Masticatory apparatus, with all its compounds and its proper growth and development, enables proper masticatory performance, and processing of a wide variety of foods [1,2].

The process of food ingestion (solid and semi-solid food) is described in four steps (4-step sequence by Hiiemae and Palmer). The food ingestion process can be stopped at each of the three checkpoints [7].

After food choice and selection, if the process passes the first checkpoint (food selection before ingestion), food is being put in the mouth. The first checkpoint is one’s acceptance or refusal of given food based on individual preferences. After selecting the food and passing the first checkpoint food portions are transported from the frontal to the lateral teeth which represents the first step of ingestion (Stage 1 Hiiemae and Palmer). The second checkpoint is the oral sensory analysis of the food. From the olfactory, gustatory, and oral receptors of the somatosensory system nerve impulses are transmitted to the cerebral cortex and truncus encephali. The cephalic phase is then activated, and the preparation of the digestive system for food digestion starts. The cephalic phase also continues during Stage II. Stage II, proper mastication, includes common activities of masticatory apparatus (soft tissues, bones, teeth, and muscles) to create a proper food bolus by placing food within the occlusal surfaces of the teeth, and with the aid of saliva. In the third stage, food bolus is being transported more orally toward the esophagus and stops at the pharyngo epiglottic folds. The second and third stage are not happening separately one after the other but they exchange cyclically, food is moving from the folds through the fauceus isthmus to the places between teeth, tongue, and cheeks to be crushed. This continues until the food bolus is properly formed which is the positive signal for the third checkpoint. Adequate food bolus, passed third checkpoint, activates Stage 4, swallowing of the food. With deglutition, food is transported from the oral cavity to the pharynx and then the esophagus [1,2,7]. 

It is evident that the food bolus properties are essential for food deglutition, controlled by the third checkpoint. Food bolus must be slippery, cohesive, and plastic which is achieved in the second and third stage of food ingestion. Chewed particle sizes are one of the determining factors for reaching the necessary properties of the bolus and consecuntelly safely swallowing the bolus. Peyron et al. Have shown that deglutition starts only when the required size of the chewed particles is being achieved [8]. To form a proper bolus with the required rheological properties individuals chew longer, increasing the number of masticatory cycles before swallowing [9]. Except for the increased number of masticatory cycles before swallowing, jaw movements and muscle bite forces can change during mastication until the second checkpoint is triggered with the right sensory input on the food bolus properties [10]. If all of these adaptive mechanisms are still not enough to crush the food into small particles and form a swallowable bolus, either too big particles are being swallowed or this type of food is avoided by the individual [7]. 

The masticatory function can be assessed by objective methods (physical measurements) and subjective methods oriented to the patient’s experience (questionnaires). The most frequently used objective methods are the measurement of muscle activity (masticatory force strength and electromyography) and the measurement of masticatory efficiency. Masticatory efficiency (ME) is defined as the individual ability to grind food, whereby a smaller surface area and a larger number of particles imply better ME. It can be measured by colorimetric and spectrophotometric techniques, scales for visual assessment, sieving, optical scanning of chewed particles, etc. [2,11].

Sieving implies fractionation of the sample through a set of sieves of precisely defined diameters and has long been considered the gold standard in the measurement of ME. However, the development of computer technology enabled the development of the method of optical particle scanning, the main advantage of which is the ability to precisely measure the dimensions and determine the shape of each particle in the sample. In addition to precision, a great advantage is the speed and simplicity of sample processing compared to the sieving technique [12]. Today, this method is considered superior to other methods [13].

Different test foods can be used to evaluate ME, most often gelatin, peanuts, carrots, and almonds. Since natural food can vary significantly in physical properties and composition and has high water content, since the 1980s, the material for impressions based on condensation silicones has been considered the standard. The advantages of silicone materials are dimensional stability, easy shaping of test samples of standard dimensions, appropriate hardness, and strength. The lack of smell and taste of silicone materials can be considered as a disadvantage compared to natural foods. However, the smell and taste of the test food can affect the patient’s mastication depending on individual preferences, and this is another reason why the silicone material chewing sample has become a standardized procedure [14].

It is believed that the premature tooth loss in children can have various negative consequences: changes in occlusion and mastication, speech disorders, development of bad oral habits, loss of space for permanent successors and consequent malocclusions, impairment of aesthetics, reduction in quality of life and altered psychological condition of the individual [15,16,17].

Neuromotor control of chewing depends on occlusal contacts between dental arches and receptors in the temporomandibular joint, periodontium, and dental pulp. Therefore, any condition that affects the structure and position of the teeth can have an impact on mastication. Masticatory efficiency can be influenced by the state of dentition, precisely the number of teeth and occlusion contact area [2]. Tooth loss leads to a decrease in masticatory efficiency (ME), and a reduced number of antagonistic contacts in the dentition (reduced surfaces of occlusal contacts), diminishing the ability to grind food [11,18]. 

There is a small number of studies that have investigated the relation of the reduced number of antagonistic contacts to ME in the younger population. In order to contribute to the understanding of the connection between these factors in the child population, this research was carried out. The aim of this study was to examine the association between ME and the reduced number of antagonistic contacts due to tooth extraction, malocclusion, and changing dentition in children.

## 2. Materials and Methods

This study included 48 children aged 3 to 14 years of both genders (24 female, 24 male) in different stages of growth and development. Participants were children with healthy dentition and those with a reduced number of antagonistic contacts due to physiological tooth exfoliation, tooth extraction, or orthodontic anomalies. The sample from the population was obtained by including patients who came for a dental visit at the Pediatric Dentistry Department of the Clinic for Dental Medicine of Clinical Hospital Center Rijeka from May 2021 to November 2022.

Only children whose parents/legal guardians gave informed consent to participate in the study were included in this research.

Participants were assigned to one of the two groups. The first group included patients with healthy dentition and preserved all antagonistic contacts. The second group included children with bilaterally lost antagonistic contacts in the posterior segment due to:

- tooth extraction due to caries (subgroup 2a),
- orthodontic anomalies (subgroup 2b)
- or dentition change (subgroup 2c).

In between the two groups, children were matched by gender and age.

Children with non-cooperative behaviour; pathological changes of the temporomandibular joint, masticatory muscles, periodontal tissues, or any other structure of the stomatognathic system were exclusion criteria for participants, as well as drugs and diseases that affect neuromuscular functions [18,19].

Sample size (*n* = 8, 4 per group) was calculated to achieve a power of 80% and a level of significance of 5%, using data from the research by Kreulen et al. (Mean 1 2.00 mm, Mean 2 2.40 mm, SD 0.20) [20]. 

This cross-sectional study used the optical scanning of particles method for the assessment of masticatory efficiency. 

Participants chewed the standardized artificial test food that was previously prepared from Optosil^®^ (Heraeus Kulzer, Hanau, Germany) according to the Protocol for standardized production of artificial test food by Albert et al. and packed in an impermeable 0.25 mm latex membrane [14]. Artificial test food, a silicon cylinder with a diameter of 20 mm and height of 5 mm, was masticated throughout 20 masticatory cycles not limiting the side of mastication or the time needed for 20 masticatory cycles. Chewing was free, it could be performed unilaterally (left or right) or bilaterally, in order to be exact as the habitual chewing, and the included subjects had a bilateral loss of occlusal contacts so that regardless of the side of mastication, the lost antagonistic contacts would be present. The time needed for 20 masticatory cycles was measured. Before evaluating the effectiveness of chewing, the entire procedure was explained to the subjects and they tried to chew one bolus substitute before the actual evaluation.

**Figure 1:**
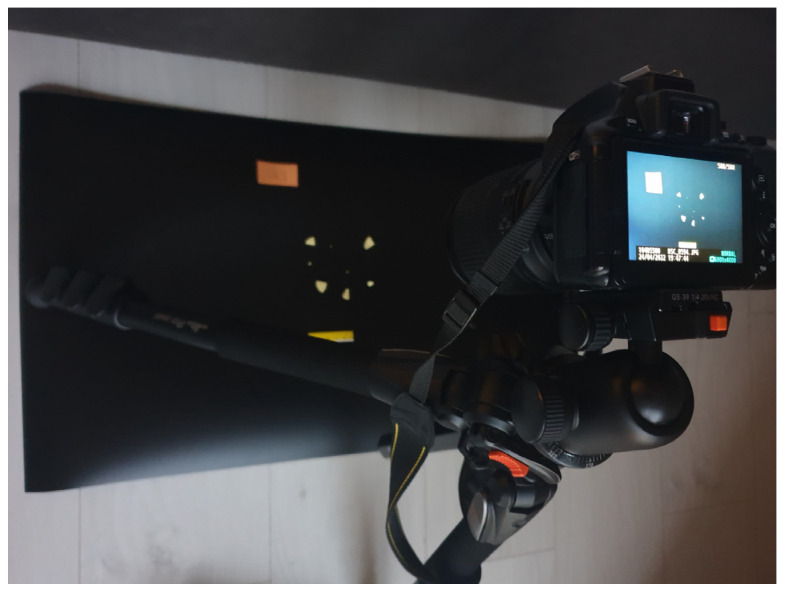
Taking standardized photographs of chewed particles.

Chewed particles were spread on a black photographing background (Figure 1). Standardized photographs of chewed particles were then analyzed with Motic Images Plus 2.0 software program (Motic, Hong Kong, China) to determine the number of particles and size (diameter and surface) of each chewed particle. A smaller mean diameter and mean surface of particles denote better ME while a smaller number of chewed particles refers to a decrease in ME.

Except for the assessment of ME via optical scanning method, each participant was examined during their dental visit. Dental status and data on aetiology and time of tooth loss were collected. Antagonistic contacts were evaluated by a 12 μm thick dental articulating paper. Also, the number of preserved and lost antagonistic contacts was determined with the recorded reason for contact loss (tooth extraction, changing dentition, orthodontic anomaly).

The research was approved by the Ethics Committee of the Clinical Hospital Center Rijeka, the Ethics Committee of the Faculty of Dental Medicine and the Ethics Committee for Biomedical Research of the Faculty of Medicine of the University of Rijeka. Also, the research is registered in ClinicalTrials.gov Database under the number NCT05000385.

### Statistical Analysis

The difference in ME parameters (number of particles, mean diameter, and mean surface) and the time interval between the two groups was tested using the Mann-Whitney test. Also, differences in ME between different aetiologies of antagonistic contact loss were tested with the Mann-Whitney test. The correlation between the number of lost antagonistic contacts and ME parameters (number of particles, mean diameter, and mean surface) was tested with Spearman’s correlation. α value of 0.05 was considered statistically significant. Statistical analysis was done using the SPSS 13.0 software (SPSS Inc., Chicago, IL, USA).

## 3. Results

There was no statistical difference in age between the two groups (Group 1 7.88 ± 2.90, Group 2 7.88 ± 2.22; *p* = 0.992 independent sample *t*-test), and groups had the same number of male (12) and female (12) participants. Data on age, gender, dentition and number of extracted teeth of participants in both groups is shown in Table 1.

The time interval for 20 masticatory cycles was not significantly different between the two groups (Group 1 = 31.98 ± 18.55 s, Group 2 = 28.83 ± 19.10 s; *p* = 0.257; Mann-Whitney test).

The number of chewed particles is significantly higher in a group of children with healthy dentition (Group 1 279.25 ± 239.82, Group 2 58.25 ± 86.56; *p* < 0.001; Mann-Whitney test), and chewed particles’ mean diameter and surface are significantly higher in Group 2 (particle diameter Group 1 2.33 ± 0.75 mm, Group 2 4.93 ± 3.57 mm; *p* < 0.001; Mann-Whitney test) (particle surface Group 1 5.86 ± 4.04 mm^2^, Group 2 23.55 ± 25.69 mm^2^; *p* < 0.01; Mann-Whitney test). 

Data and descriptive statistics on the number and size of chewed particles are shown in Table 2. 

Participants in Group 2 had on average 5.83 ± 2.55 missing antagonistic contacts (min. 2, max. 12). There is no statistically significant correlation between the number of antagonistic contacts and masticatory efficiency; particles number, diameter, and surface (*p*= 0.464; *p*= 0.483; *p*= 0.489). Also, there is no correlation between the time interval and the number of antagonistic contacts (*p* = 0.079). 

**Figure 2:**
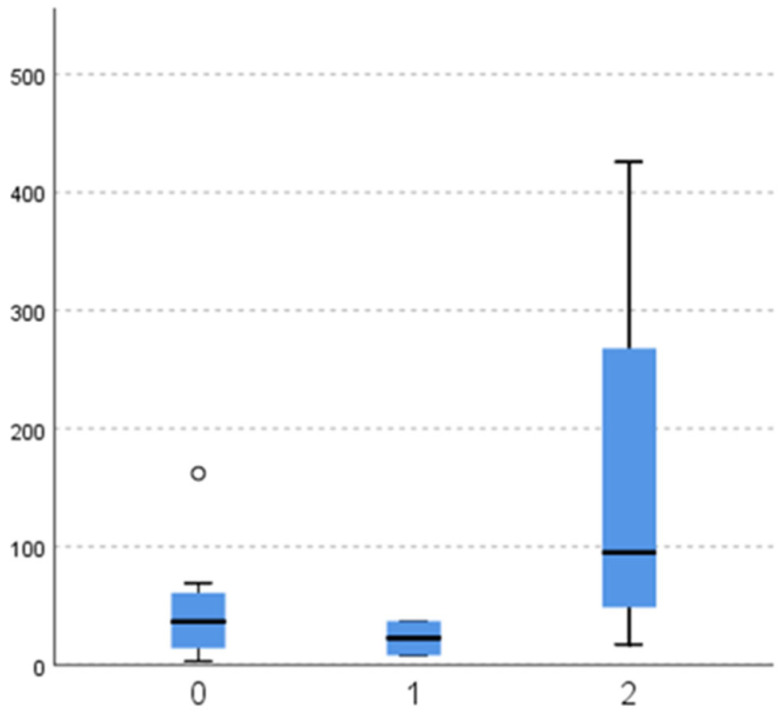
Number of chewed particles (y-axis) according to the aetiology of antagonistic contact loss (x-axis: 0—tooth extraction (subgroup 2a), 1—malocclusion (2b), 2—changing dentition (2c)).

**Figure 3:**
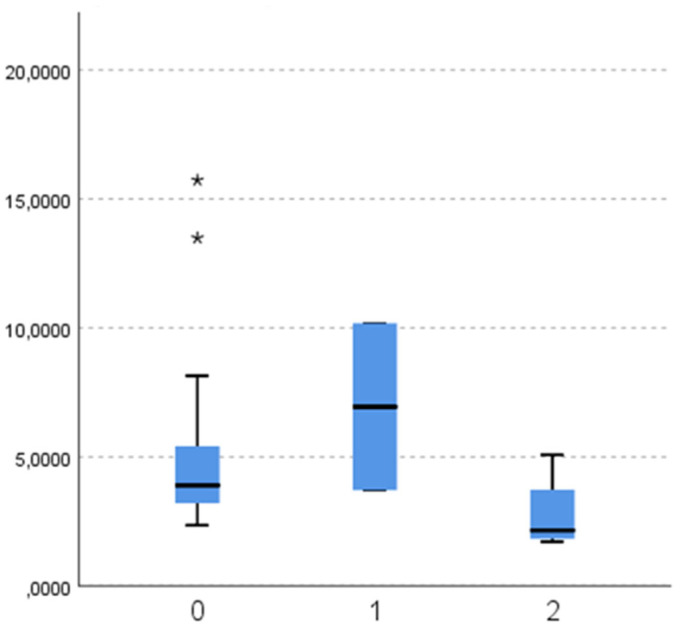
Diameter (mm) of chewed particles (y-axis) according to the aetiology of antagonistic contact loss (x-axis: 0—tooth extraction (subgroup 2a), 1—malocclusion (2b), 2—changing dentition (2c)). (*-outliers).

**Figure 4:**
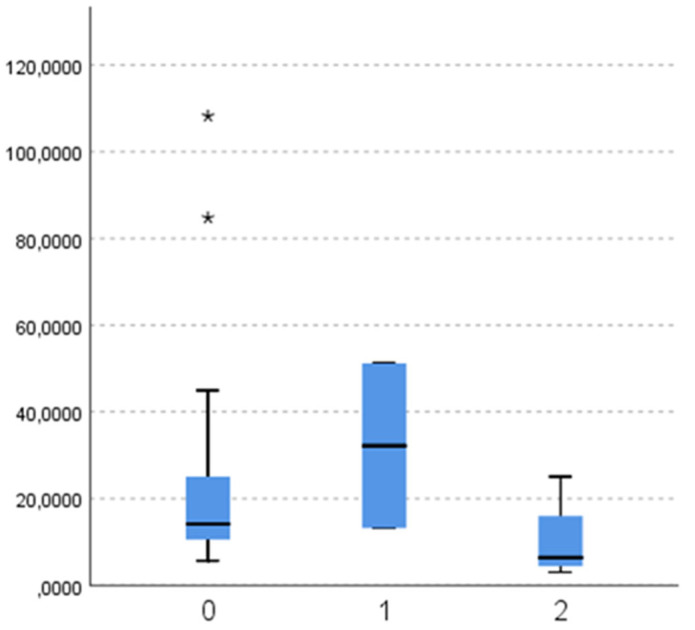
Surface (mm^2^) of chewed particles (y-axis) according to the aetiology of antagonistic contact loss (x-axis: 0—tooth extraction (subgroup 2a), 1—malocclusion (2b), 2—changing dentition (2c)). (*-outliers).

When comparing masticatory efficiency variables between different aetiologies of lost antagonistic contact, there is no significant difference in particle number (Figure 2; *p* = 0.121), diameter (Figure 3; *p* = 0.121), and surface (Figure 4; *p* = 0.139). 

Values of ME variables in Group 2 according to the aetiology of antagonistic contact loss are presented in Table 3.

## 4. Discussion

This research showed decreased masticatory efficiency (smaller number, and bigger mean diameter and surface of chewed particles) in a group of children with a decrease in the number of occluding teeth (Group 2). 

The Masticatory Normative index, MNI is defined as a chewed particle diameter of 4 mm. If the chewed particles’ diameter is 4 mm or smaller it is considered that the masticatory function is adequate [7]. The mean particle diameter of chewed particles (4.93 mm) is bigger than the MNI in a group of participants with antagonistic contact loss. While the mean particle diameter (2.33 mm) in Group 1, children with healthy dentition and present all antagonistic contacts, is smaller than the MNI. This suggests that the mastication is adequate in Group 1 and impaired in Group 2. 

Bigger chewed particle diameter in participants with less occluding teeth can be compensated by a bigger number of masticatory cycles, masticatory force change, and jaw movement change. With the bigger number of masticatory cycles before swallowing children with loss antagonistic tooth contacts can potentially reach a mean particle diameter smaller than 4 mm, achieve adequate plasticity, cohesiveness, and slipperiness of food bolus, and still have an unobstructed start of digestive function. But, if they do not increase the number of masticatory cycles sufficiently and do not reach a diameter of less than 4 mm, meaning that the food bolus does not reach requirements for deglutition their masticatory and consequently digestive function is impaired [7]. Reduced chewing efficiency (larger chewed particle sizes) results in changing one’s dietary habits (which can lead to nutritional imbalance) or the digestive system will have to process larger food particles, which can lead to digestive disorders and decrease intestinal absorption of nutrients. This means that in addition to changes in dietary habits, reduced absorption of food, and nutritional deficit, a reduced ability to break down food can lead to disorders of the digestive system, such as obstruction of the esophagus, disorders in esophageal peristalsis, slowed gastric emptying, more severe forms of chronic gastritis and H. pylori infection in patients with dyspepsia, etc [21,22]. Many studies have proven that impaired chewing efficiency leads to inadequate food choices. Inadequate food choices, adapting food choices to impaired masticatory function, includes reducing consumption of foods that are more difficult to chew and increasing consumption of foods that are easier to chew. Reduced consumption of foods that are more difficult to chew in people with impaired chewing efficiency means avoiding hard crunchy foods such as raw vegetables and fruits, tough fibrous foods such as meat, and dry hard foods such as whole grains and nuts. The above-mentioned foods belong to the group of foods with high nutritional density and are the main source of protein, unrefined carbohydrates, dietary fiber, vitamins, and minerals. Furthermore, people with reduced masticatory efficiency consume more industrially processed food, which is softer and easier to chew. Such food has a high proportion of saturated fatty acids, refined carbohydrates, salt, and sugar. Also, high processing which makes food easier to chew can reduce the amount of essential nutrients—vitamins and minerals [21,23,24,25,26,27]. According to research by Krall et al. intake of fiber, vitamins (A, C, B1, B2, B6, B9, D), and minerals (Mg, P, Fe) positively correlates with chewing efficiency [23]. 

Previous studies have shown that the masticatory efficiency is in correlation with the number of occluding teeth [28,29], and is impaired when there are less than 20 occluding units [28,30]. But, results from this study do not support this idea; there was no significant correlation between masticatory efficiency and the number of lost occlusal contacts. Other studies have shown that masticatory ability is not just influenced by the number of occluding teeth, but also by the type and distribution of remaining occluding teeth. Studies have shown that masticatory function is more reduced when there are fewer occluding premolar teeth and/or when the remaining teeth are placed asymmetrically in dental arches; while there is no big impact on masticatory ability when the dental arch is shortened and occlusal contacts in premolar regions are preserved [29,31]. The posterior functional unit (PFU) is defined as a number of posterior occluding pairs of teeth. Its maximum value is 8 (4 premolar and 4 molar occluding pairs) or 10 in the case of the presence of wisdom teeth, and it can be used for masticatory performance examination [7]. But, for this study, whose participants are children with primary or changing dentition, PFU can not be applied because of a smaller total number of occluding pairs in the primary and changing dentition.

These results on reduced masticatory performance in participants with missing occluding units are supported by other studies that were conducted on adult participants and that have shown a statistically significant increase in chewed particle sizes in participants with a fewer number of occluding teeth [32,33,34]. Studies on ME with a reduced number of occluding units in the adult population investigated ME after tooth loss/tooth extraction. Except for tooth extraction, antagonistic contacts of posterior teeth can be lost due to malocclusion or physiological tooth exfoliation during changing dentition in the pediatric population.

Orthodontic anomalies are considered one of the factors that can affect the masticatory process. A lower ME has been demonstrated in patients with an open or crossbite compared to patients with normoocclusion, and in patients with Angle class II or III compared to class I patients [35,36]. In the pediatric population, a unilateral crossbite is a malocclusion that is considered to have the greatest impact on ME [3]. The reason why malocclusions can potentially impair ME is not fully clarified. It may be related to the muscular and bony changes of the stomatognathic system associated with malocclusions, or it may be caused by a reduction in the area of occlusal contacts due to malocclusions. This study showed that children with malocclusion (that causes loss of occlusal contacts) have a ME that is no different from the children with extracted teeth. Moreover, the mean chewed particle diameter of 6.94 mm is higher than the MNI which means that children with a reduced number of antagonistic tooth contacts have impaired mastication.

In addition, the question arises about the ME during physiological changes—tooth exfoliation and eruption when all antagonistic tooth contacts are not present but there are no pathological changes in the stomatognathic system. Does the reduced number of antagonistic contacts during changing dentition correlate with reduced ME or does the stomatognathic system adapt to this physiological process? In this research, there was no difference in ME of participants with contact loss due to changing dentition in comparison to ME in children with antagonistic tooth contact missing due to extraction or missing occlusal contacts due to malocclusion. However, when analyzing the chewed particle sizes in Group 2 it is evident that children with a decreased number of occluding teeth due to pathological changes (tooth extraction and orthodontic anomalies) have a mean chewed particle diameter higher than the MNI (4.87 mm, 6.94 mm) while children with contact loss due to changing dentition have a mean chewed particle diameter smaller than the MNI (2.77 mm). So, although there was no significant difference in ME between different aetiologies when looking at the mean chewed particle diameter it is evident that children in a phase of changing dentition have adequate mastication while children with extracted teeth and orthodontic anomalies that reduce the number of occluding teeth have impaired mastication. Once again, children with extracted teeth and orthodontic anomalies that reduce the number of occluding teeth can compensate for this reduced chewing efficiency in several ways. These findings suggest that tooth exfoliation and eruption, as physiological processes, do not need compensatory changes to achieve proper food boluses. 

The main limitation of the study was the small number of participants for the ME comparison in Group 2 based on different aetiologies. Therefore, further studies should be conducted on ME in children with a reduced number of antagonistic contacts, but with taking into account different aetiologies of contact loss.

## 5. Conclusions

To conclude, children with a reduced number of antagonistic contacts have a significantly reduced masticatory performance in comparison to children with full healthy dentition. Reduced ME is not in correlation with the number of antagonistic contacts and does not differentiate between different causes of contact loss.

## Figures and Tables

**Table 1:** Age, gender, dentition and number of participants.

		Group 1	Group 2
		Complete Healthy Dentition	Reduced Number of Antagonistic Contacts
Number of Participants (Male, Female)		24 (12 M, 12 F)	24 (12 M, 12 F)
Number of Participants	deciduous dentition	9	9
	mixed dentition	13	13
	permanent dentition	2	2
Number of extracted teeth	permanent	0	82
	permanent	0	8

**Table 2:** Masticatory efficiency—particle number and sizes.

	Number of Particles				Particle Diameter (mm)				Particle Surface (mm^2^)			
	Min.	Max.	Mean	SD	Min.	Max.	Mean	SD	Min.	Max.	Mean	SD
Group 1 (*n* = 24)	27	1000	279.25	239.82	1.49	4.02	2.33	0.75	2.40	15.51	5.86	4.04
Group 2 (*n* = 24)	3	426	58.25	86.56	1.71	15.73	4.93	3.57	3.1	108.21	23.55	25.69

**Table 3:** Number, mean diameter, and mean surface of chewed particles, and the time interval for 20 masticatory cycles in participants with different aetiologies of antagonistic contact loss.

Contact Loss Aetiology	Particle Number		Particle Diameter (mm)		Particle Surface (mm^2^)		Time Interval (s)	
	Mean	SD	Mean	SD	Mean	SD	Mean	SD
tooth extraction (2a) *n* = 15	48.05	43.01	4.87	3.67	23.53	27.10	27.48	14.95
orthodontic anomaly (2b) *n* = 4	22.5	20.06	6.94	4.58	32.19	26.86	63.19	43.37
changing dentition (2c) *n* = 5	158.25	182.66	2.77	1.57	10.22	10.04	22.27	8.29

## Data Availability

The data is not publicly available.
